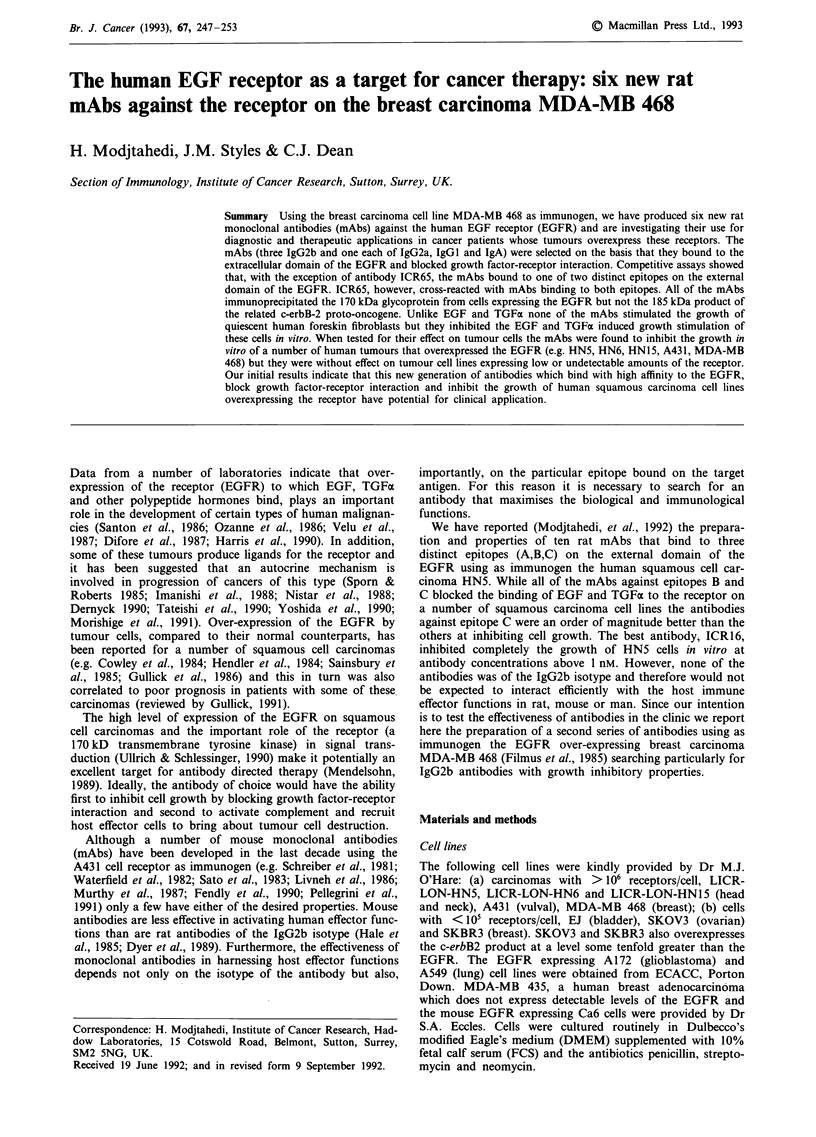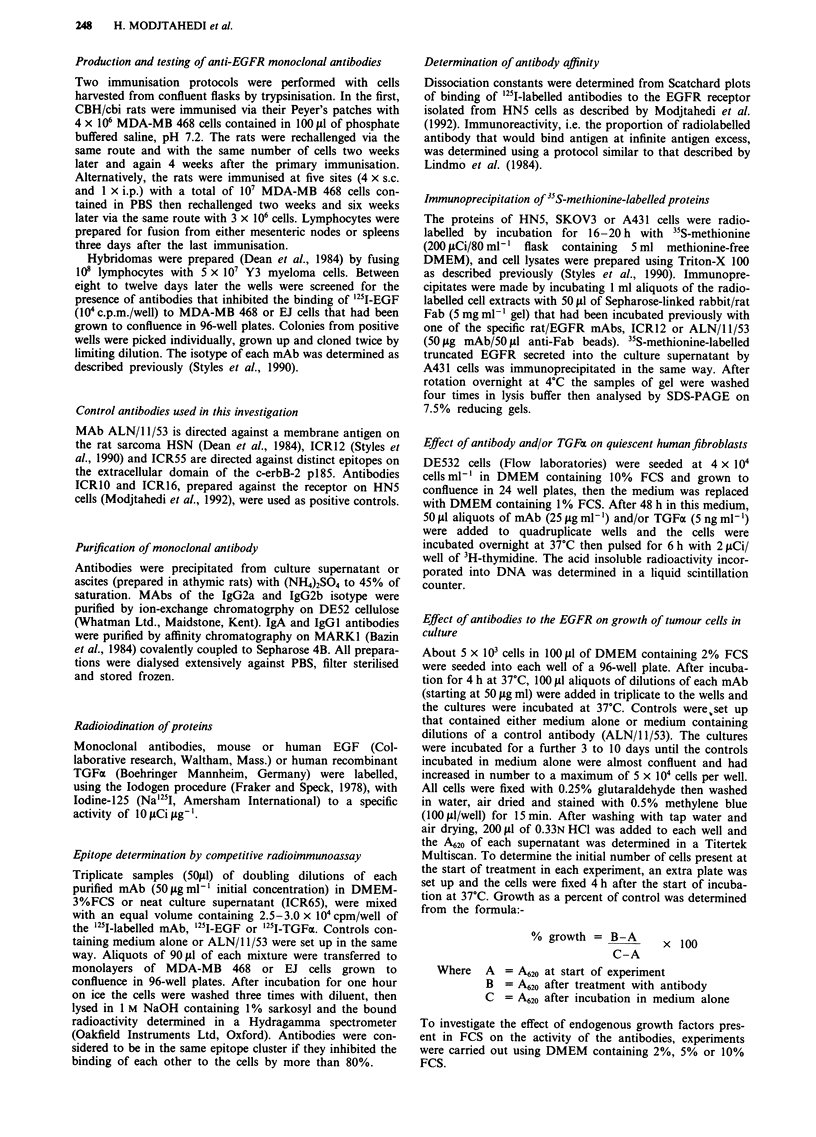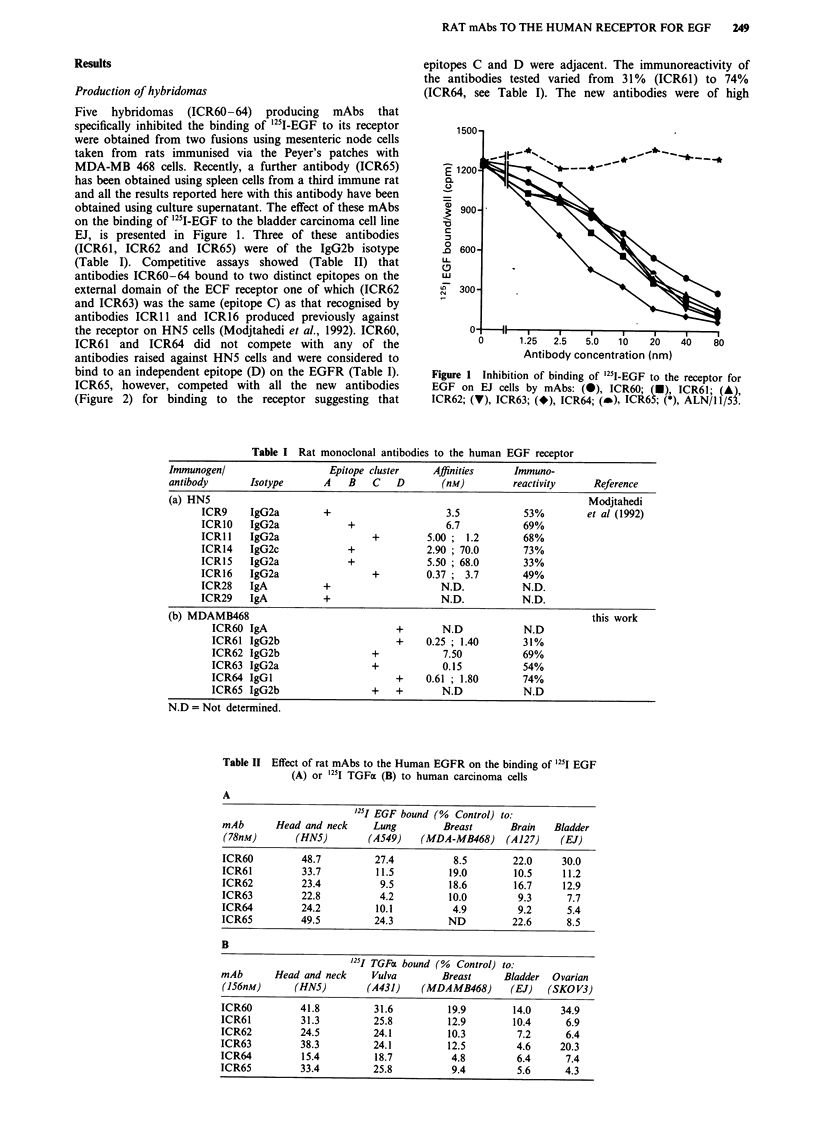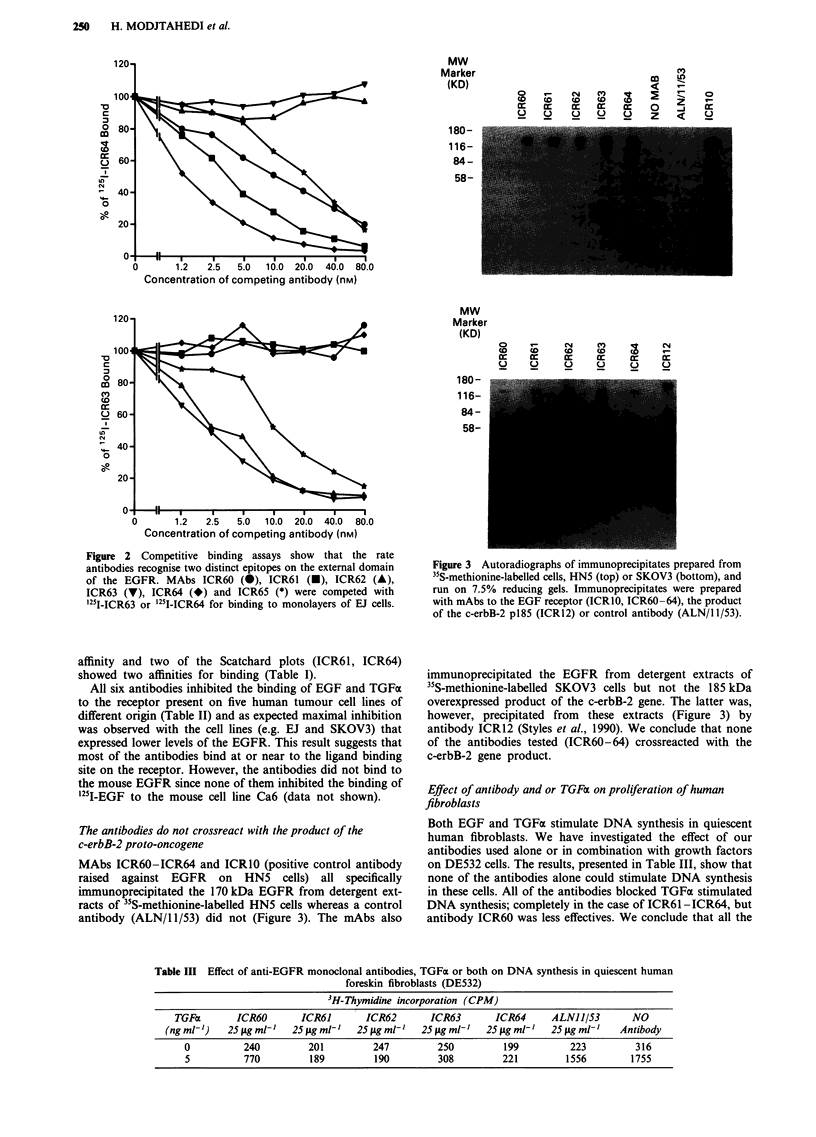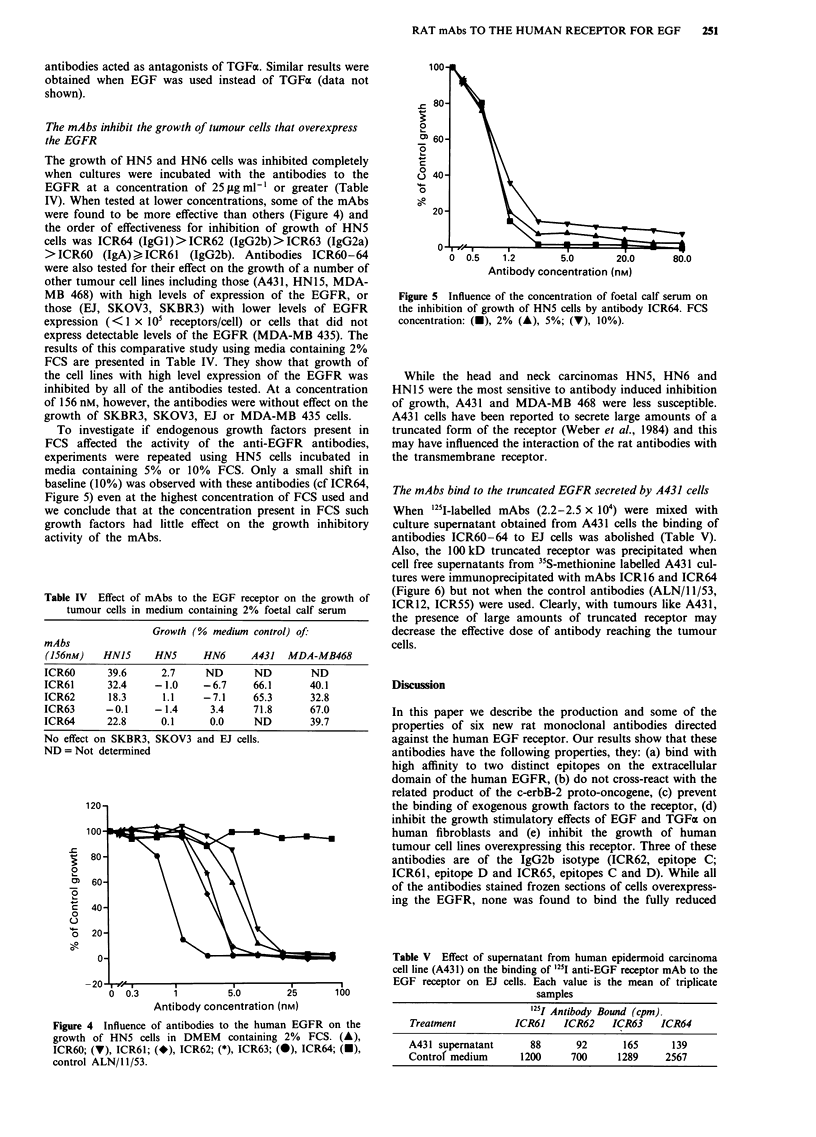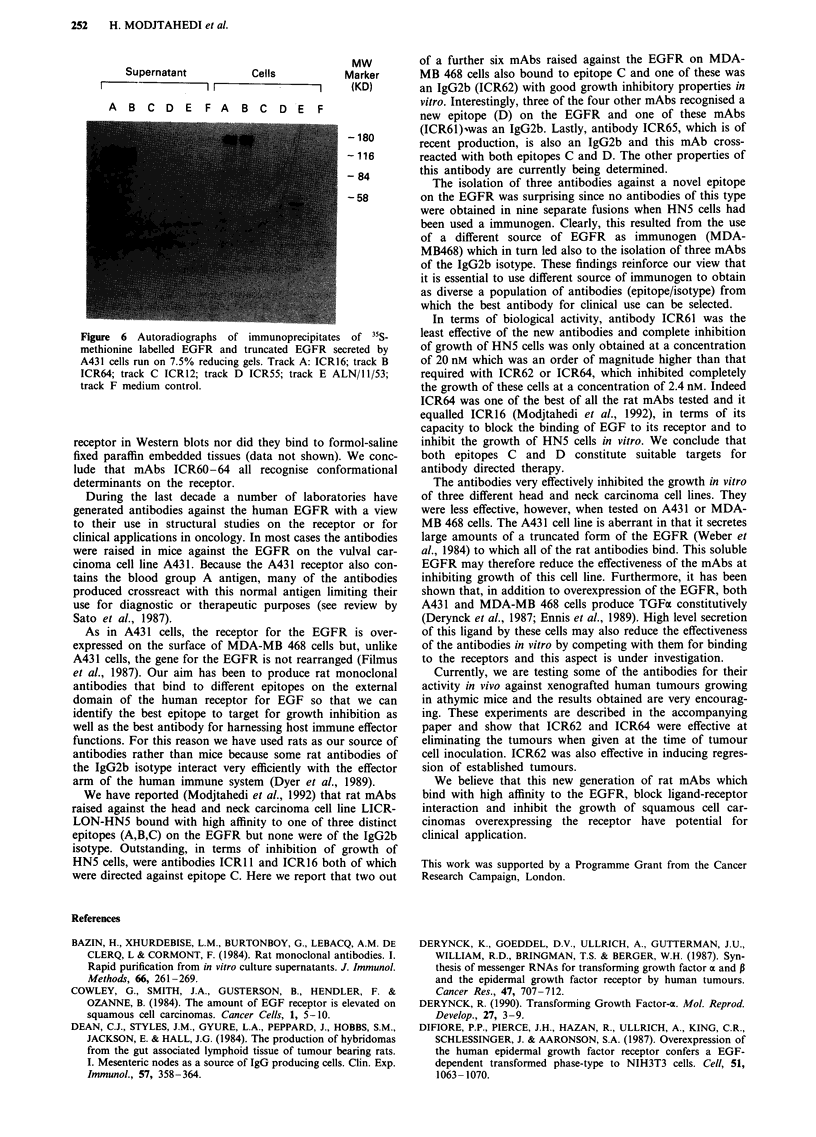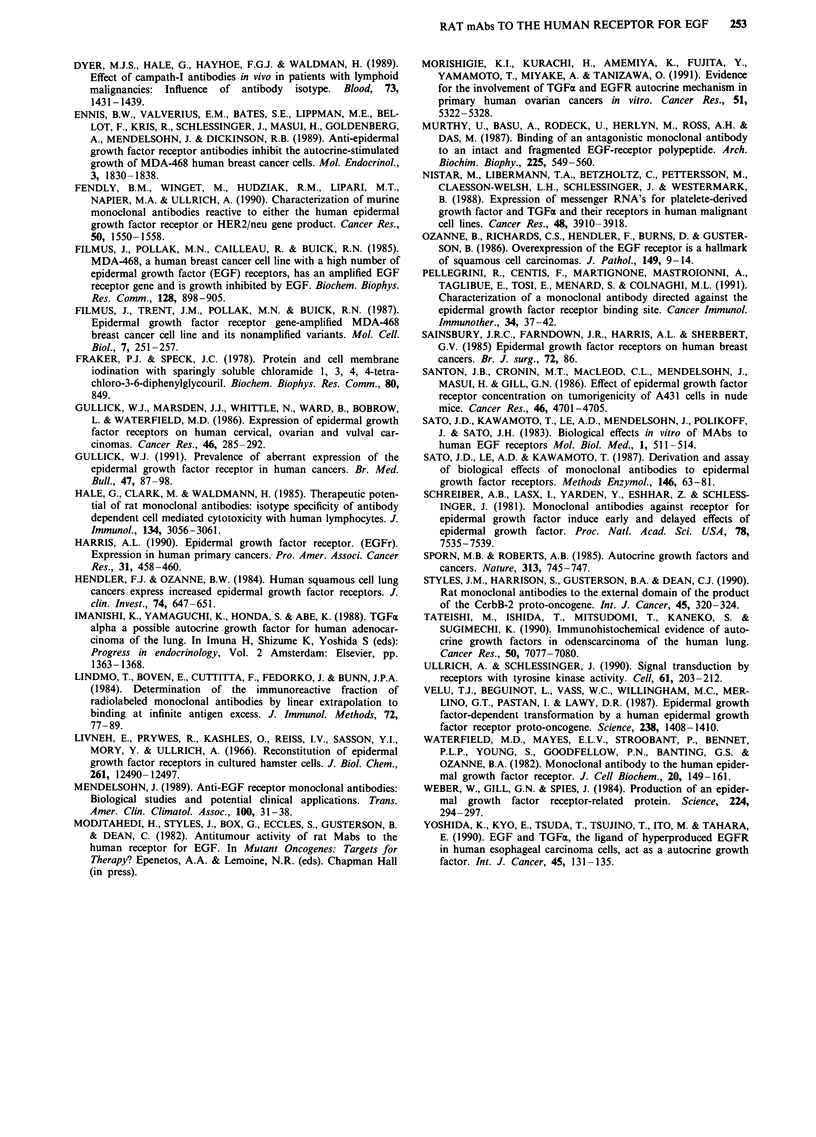# The human EGF receptor as a target for cancer therapy: six new rat mAbs against the receptor on the breast carcinoma MDA-MB 468.

**DOI:** 10.1038/bjc.1993.48

**Published:** 1993-02

**Authors:** H. Modjtahedi, J. M. Styles, C. J. Dean

**Affiliations:** Section of Immunology, Institute of Cancer Research, Sutton, Surrey, UK.

## Abstract

**Images:**


Br. J. Cancer (1993), **67**, 247–253 © Macmillan Press Ltd., 1993

### The human EGF receptor as a target for cancer therapy: six new rat mAbs against the receptor on the breast carcinoma MDA-MB 468

H. Modjtahedi, J.M. Styles & C.J. Dean

*Section of Immunology, Institute of Cancer Research, Sutton, Surrey, UK.*

**Summary** Using the breast carcinoma cell line MDA-MB 468 as immunogen, we have produced six new rat monoclonal antibodies (mAbs) against the human EGF receptor (EGFR) and are investigating their use for diagnostic and therapeutic applications in cancer patients whose tumours overexpress these receptors. The mAbs (three IgG2b and one each of IgG2a, IgG1 and IgA) were selected on the basis that they bound to the extracellular domain of the EGFR and blocked growth factor-receptor interaction. Competitive assays showed that, with the exception of antibody ICR65, the mAbs bound to one of two distinct epitopes on the external domain of the EGFR. ICR65, however, cross-reacted with mAbs binding to both epitopes. All of the mAbs immunoprecipitated the 170 kDa glycoprotein from cells expressing the EGFR but not the 185 kDa product of the related c-erbB-2 proto-oncogene. Unlike EGF and TGFα none of the mAbs stimulated the growth of quiescent human foreskin fibroblasts but they inhibited the EGF and TGFα induced growth stimulation of these cells *in vitro*. When tested for their effect on tumour cells the mAbs were found to inhibit the growth *in vitro* of a number of human tumours that overexpressed the EGFR (e.g. HN5, HN6, HN15, A431, MDA-MB 468) but they were without effect on tumour cell lines expressing low or undetectable amounts of the receptor. Our initial results indicate that this new generation of antibodies which bind with high affinity to the EGFR, block growth factor-receptor interaction and inhibit the growth of human squamous carcinoma cell lines overexpressing the receptor have potential for clinical application.

Data from a number of laboratories indicate that over-expression of the receptor (EGFR) to which EGF, TGFα and other polypeptide hormones bind, plays an important role in the development of certain types of human malignancies (Santon *et al.*, 1986; Ozanne *et al.*, 1986; Velu *et al.*, 1987; Difore *et al.*, 1987; Harris *et al.*, 1990). In addition, some of these tumours produce ligands for the receptor and it has been suggested that an autocrine mechanism is involved in progression of cancers of this type (Sporn & Roberts 1985; Imanishi *et al.*, 1988; Nistar *et al.*, 1988; Dernyck 1990; Tateishi *et al.*, 1990; Yoshida *et al.*, 1990; Morishige *et al.*, 1991). Over-expression of the EGFR by tumour cells, compared to their normal counterparts, has been reported for a number of squamous cell carcinomas (e.g. Cowley *et al.*, 1984; Hendler *et al.*, 1984; Sainsbury *et al.*, 1985; Gullick *et al.*, 1986) and this in turn was also correlated to poor prognosis in patients with some of these carcinomas (reviewed by Gullick, 1991).

The high level of expression of the EGFR on squamous cell carcinomas and the important role of the receptor (a 170 kD transmembrane tyrosine kinase) in signal transduction (Ullrich & Schlessinger, 1990) make it potentially an excellent target for antibody directed therapy (Mendelsohn, 1989). Ideally, the antibody of choice would have the ability first to inhibit cell growth by blocking growth factor-receptor interaction and second to activate complement and recruit host effector cells to bring about tumour cell destruction.

Although a number of mouse monoclonal antibodies (mAbs) have been developed in the last decade using the A431 cell receptor as immunogen (e.g. Schreiber *et al.*, 1981; Waterfield *et al.*, 1982; Sato *et al.*, 1983; Livneh *et al.*, 1986; Murthy *et al.*, 1987; Fendly *et al.*, 1990; Pellegrini *et al.*, 1991) only a few have either of the desired properties. Mouse antibodies are less effective in activating human effector functions than are rat antibodies of the IgG2b isotype (Hale *et al.*, 1985; Dyer *et al.*, 1989). Furthermore, the effectiveness of monoclonal antibodies in harnessing host effector functions depends not only on the isotype of the antibody but also, importantly, on the particular epitope bound on the target antigen. For this reason it is necessary to search for an antibody that maximises the biological and immunological functions.

We have reported (Modjtahedi, *et al.*, 1992) the preparation and properties of ten rat mAbs that bind to three distinct epitopes (A,B,C) on the external domain of the EGFR using as immunogen the human squamous cell carcinoma HN5. While all of the mAbs against epitopes B and C blocked the binding of EGF and TGFα to the receptor on a number of squamous carcinoma cell lines the antibodies against epitope C were an order of magnitude better than the others at inhibiting cell growth. The best antibody, ICR16, inhibited completely the growth of HN5 cells *in vitro* at antibody concentrations above 1 nM. However, none of the antibodies was of the IgG2b isotype and therefore would not be expected to interact efficiently with the host immune effector functions in rat, mouse or man. Since our intention is to test the effectiveness of antibodies in the clinic we report here the preparation of a second series of antibodies using as immunogen the EGFR over-expressing breast carcinoma MDA-MB 468 (Filmus *et al.*, 1985) searching particularly for IgG2b antibodies with growth inhibitory properties.

### Materials and methods

### *Cell lines*

The following cell lines were kindly provided by Dr M.J. O'Hare: (a) carcinomas with $>10^6$ receptors/cell, LICR-LON-HN5, LICR-LON-HN6 and LICR-LON-HN15 (head and neck), A431 (vulval), MDA-MB 468 (breast); (b) cells with $<10^5$ receptors/cell, EJ (bladder), SKOV3 (ovarian) and SKBR3 (breast). SKOV3 and SKBR3 also overexpresses the c-*erb*B2 product at a level some tenfold greater than the EGFR. The EGFR expressing A172 (glioblastoma) and A549 (lung) cell lines were obtained from ECACC, Porton Down. MDA-MB 435, a human breast adenocarcinoma which does not express detectable levels of the EGFR and the mouse EGFR expressing Ca6 cells were provided by Dr S.A. Eccles. Cells were cultured routinely in Dulbecco's modified Eagle's medium (DMEM) supplemented with 10% fetal calf serum (FCS) and the antibiotics penicillin, streptomycin and neomycin.

Correspondence: H. Modjtahedi, Institute of Cancer Research, Haddow Laboratories, 15 Cotswold Road, Belmont, Sutton, Surrey, SM2 5NG, UK.
Received 19 June 1992; and in revised form 9 September 1992.

*Production and testing of anti-EGFR monoclonal antibodies*

Two immunisation protocols were performed with cells harvested from confluent flasks by trypsinisation. In the first, CBH/cbi rats were immunised via their Peyer's patches with $4 \times 10^6$ MDA-MB 468 cells contained in 100 µl of phosphate buffered saline, pH 7.2. The rats were rechallenged via the same route and with the same number of cells two weeks later and again 4 weeks after the primary immunisation. Alternatively, the rats were immunised at five sites (4 × s.c. and 1 × i.p.) with a total of $10^7$ MDA-MB 468 cells contained in PBS then rechallenged two weeks and six weeks later via the same route with $3 \times 10^6$ cells. Lymphocytes were prepared for fusion from either mesenteric nodes or spleens three days after the last immunisation.

Hybridomas were prepared (Dean *et al.*, 1984) by fusing $10^8$ lymphocytes with $5 \times 10^7$ Y3 myeloma cells. Between eight to twelve days later the wells were screened for the presence of antibodies that inhibited the binding of $^{125}$I-EGF ($10^4$ c.p.m./well) to MDA-MB 468 or EJ cells that had been grown to confluence in 96-well plates. Colonies from positive wells were picked individually, grown up and cloned twice by limiting dilution. The isotype of each mAb was determined as described previously (Styles *et al.*, 1990).

*Control antibodies used in this investigation*

MAb ALN/11/53 is directed against a membrane antigen on the rat sarcoma HSN (Dean *et al.*, 1984), ICR12 (Styles *et al.*, 1990) and ICR55 are directed against distinct epitopes on the extracellular domain of the c-erbB-2 p185. Antibodies ICR10 and ICR16, prepared against the receptor on HN5 cells (Modjtahedi *et al.*, 1992), were used as positive controls.

*Purification of monoclonal antibody*

Antibodies were precipitated from culture supernatant or ascites (prepared in athymic rats) with $(NH_4)_2SO_4$ to 45% of saturation. MAbs of the IgG2a and IgG2b isotype were purified by ion-exchange chromatogrphy on DE52 cellulose (Whatman Ltd., Maidstone, Kent). IgA and IgG1 antibodies were purified by affinity chromatography on MARK1 (Bazin *et al.*, 1984) covalently coupled to Sepharose 4B. All preparations were dialysed extensively against PBS, filter sterilised and stored frozen.

*Radioiodination of proteins*

Monoclonal antibodies, mouse or human EGF (Collaborative research, Waltham, Mass.) or human recombinant TGFα (Boehringer Mannheim, Germany) were labelled, using the Iodogen procedure (Fraker and Speck, 1978), with Iodine-125 (Na$^{125}$I, Amersham International) to a specific activity of 10 µCi µg$^{-1}$.

*Epitope determination by competitive radioimmunoassay*

Triplicate samples (50µl) of doubling dilutions of each purified mAb (50 µg ml$^{-1}$ initial concentration) in DMEM-3%FCS or neat culture supernatant (ICR65), were mixed with an equal volume containing $2.5-3.0 \times 10^4$ cpm/well of the $^{125}$I-labelled mAb, $^{125}$I-EGF or $^{125}$I-TGFα. Controls containing medium alone or ALN/11/53 were set up in the same way. Aliquots of 90 µl of each mixture were transferred to monolayers of MDA-MB 468 or EJ cells grown to confluence in 96-well plates. After incubation for one hour on ice the cells were washed three times with diluent, then lysed in 1 M NaOH containing 1% sarkosyl and the bound radioactivity determined in a Hydragamma spectrometer (Oakfield Instruments Ltd, Oxford). Antibodies were considered to be in the same epitope cluster if they inhibited the binding of each other to the cells by more than 80%.

*Determination of antibody affinity*

Dissociation constants were determined from Scatchard plots of binding of $^{125}$I-labelled antibodies to the EGFR receptor isolated from HN5 cells as described by Modjtahedi *et al.* (1992). Immunoreactivity, i.e. the proportion of radiolabelled antibody that would bind antigen at infinite antigen excess, was determined using a protocol similar to that described by Lindmo *et al.* (1984).

*Immunoprecipitation of $^{35}$S-methionine-labelled proteins*

The proteins of HN5, SKOV3 or A431 cells were radiolabelled by incubation for 16–20 h with $^{35}$S-methionine (200 µCi/80 ml$^{-1}$ flask containing 5 ml methionine-free DMEM), and cell lysates were prepared using Triton-X 100 as described previously (Styles *et al.*, 1990). Immunoprecipitates were made by incubating 1 ml aliquots of the radiolabelled cell extracts with 50 µl of Sepharose-linked rabbit/rat Fab (5 mg ml$^{-1}$ gel) that had been incubated previously with one of the specific rat/EGFR mAbs, ICR12 or ALN/11/53 (50 µg mAb/50 µl anti-Fab beads). $^{35}$S-methionine-labelled truncated EGFR secreted into the culture supernatant by A431 cells was immunoprecipitated in the same way. After rotation overnight at 4°C the samples of gel were washed four times in lysis buffer then analysed by SDS-PAGE on 7.5% reducing gels.

*Effect of antibody and/or TGFα on quiescent human fibroblasts*

DE532 cells (Flow laboratories) were seeded at $4 \times 10^4$ cells ml$^{-1}$ in DMEM containing 10% FCS and grown to confluence in 24 well plates, then the medium was replaced with DMEM containing 1% FCS. After 48 h in this medium, 50 µl aliquots of mAb (25 µg ml$^{-1}$) and/or TGFα (5 ng ml$^{-1}$) were added to quadruplicate wells and the cells were incubated overnight at 37°C then pulsed for 6 h with 2 µCi/well of $^3$H-thymidine. The acid insoluble radioactivity incorporated into DNA was determined in a liquid scintillation counter.

*Effect of antibodies to the EGFR on growth of tumour cells in culture*

About $5 \times 10^3$ cells in 100 µl of DMEM containing 2% FCS were seeded into each well of a 96-well plate. After incubation for 4 h at 37°C, 100 µl aliquots of dilutions of each mAb (starting at 50 µg ml) were added in triplicate to the wells and the cultures were incubated at 37°C. Controls were set up that contained either medium alone or medium containing dilutions of a control antibody (ALN/11/53). The cultures were incubated for a further 3 to 10 days until the controls incubated in medium alone were almost confluent and had increased in number to a maximum of $5 \times 10^4$ cells per well. All cells were fixed with 0.25% glutaraldehyde then washed in water, air dried and stained with 0.5% methylene blue (100 µl/well) for 15 min. After washing with tap water and air drying, 200 µl of 0.33N HCl was added to each well and the $A_{620}$ of each supernatant was determined in a Titertek Multiscan. To determine the initial number of cells present at the start of treatment in each experiment, an extra plate was set up and the cells were fixed 4 h after the start of incubation at 37°C. Growth as a percent of control was determined from the formula:-

$$\% \text{ growth} = \frac{B-A}{C-A} \times 100$$

Where A = $A_{620}$ at start of experiment
B = $A_{620}$ after treatment with antibody
C = $A_{620}$ after incubation in medium alone

To investigate the effect of endogenous growth factors present in FCS on the activity of the antibodies, experiments were carried out using DMEM containing 2%, 5% or 10% FCS.

### Results

### *Production of hybridomas*

Five hybridomas (ICR60–64) producing mAbs that specifically inhibited the binding of $^{125}$I-EGF to its receptor were obtained from two fusions using mesenteric node cells taken from rats immunised via the Peyer's patches with MDA-MB 468 cells. Recently, a further antibody (ICR65) has been obtained using spleen cells from a third immune rat and all the results reported here with this antibody have been obtained using culture supernatant. The effect of these mAbs on the binding of $^{125}$I-EGF to the bladder carcinoma cell line EJ, is presented in Figure 1. Three of these antibodies (ICR61, ICR62 and ICR65) were of the IgG2b isotype (Table I). Competitive assays showed (Table II) that antibodies ICR60–64 bound to two distinct epitopes on the external domain of the ECF receptor one of which (ICR62 and ICR63) was the same (epitope C) as that recognised by antibodies ICR11 and ICR16 produced previously against the receptor on HN5 cells (Modjtahedi *et al.*, 1992). ICR60, ICR61 and ICR64 did not compete with any of the antibodies raised against HN5 cells and were considered to bind to an independent epitope (D) on the EGFR (Table I). ICR65, however, competed with all the new antibodies (Figure 2) for binding to the receptor suggesting that epitopes C and D were adjacent. The immunoreactivity of the antibodies tested varied from 31% (ICR61) to 74% (ICR64, see Table I). The new antibodies were of high

**Figure 1** Inhibition of binding of $^{125}$I-EGF to the receptor for EGF on EJ cells by mAbs: (●), ICR60; (■), ICR61; (▲), ICR62; (▼), ICR63; (◆), ICR64; (♣), ICR65; (*), ALN/11/53.

**Table I** Rat monoclonal antibodies to the human EGF receptor

| *Immunogen/ antibody* | *Isotype* | *Epitope cluster A* | *B* | *C* | *D* | *Affinities (nM)* | *Immuno-reactivity* | *Reference* |
|---|---|---|---|---|---|---|---|---|
| (a) HN5 | | | | | | | | Modjtahedi *et al* (1992) |
| ICR9 | IgG2a | + | | | | 3.5 | 53% | |
| ICR10 | IgG2a | | + | | | 6.7 | 69% | |
| ICR11 | IgG2a | | | + | | 5.00 ; 1.2 | 68% | |
| ICR14 | IgG2c | | + | | | 2.90 ; 70.0 | 73% | |
| ICR15 | IgG2a | | + | | | 5.50 ; 68.0 | 33% | |
| ICR16 | IgG2a | | | + | | 0.37 ; 3.7 | 49% | |
| ICR28 | IgA | + | | | | N.D. | N.D. | |
| ICR29 | IgA | + | | | | N.D. | N.D. | |
| (b) MDAMB468 | | | | | | | | this work |
| ICR60 | IgA | | | | + | N.D | N.D | |
| ICR61 | IgG2b | | | | + | 0.25 ; 1.40 | 31% | |
| ICR62 | IgG2b | | | + | | 7.50 | 69% | |
| ICR63 | IgG2a | | | + | | 0.15 | 54% | |
| ICR64 | IgG1 | | | | + | 0.61 ; 1.80 | 74% | |
| ICR65 | IgG2b | | | + | + | N.D | N.D | |

N.D = Not determined.

**Table II** Effect of rat mAbs to the Human EGFR on the binding of $^{125}$I EGF (**A**) or $^{125}$I TGFα (**B**) to human carcinoma cells

**A**

| *mAb (78nM)* | *$^{125}$I EGF bound (% Control) to:* Head and neck (HN5) | *Lung (A549)* | *Breast (MDA-MB468)* | *Brain (A127)* | *Bladder (EJ)* |
|---|---|---|---|---|---|
| ICR60 | 48.7 | 27.4 | 8.5 | 22.0 | 30.0 |
| ICR61 | 33.7 | 11.5 | 19.0 | 10.5 | 11.2 |
| ICR62 | 23.4 | 9.5 | 18.6 | 16.7 | 12.9 |
| ICR63 | 22.8 | 4.2 | 10.0 | 9.3 | 7.7 |
| ICR64 | 24.2 | 10.1 | 4.9 | 9.2 | 5.4 |
| ICR65 | 49.5 | 24.3 | ND | 22.6 | 8.5 |

**B**

| *mAb (156nM)* | *$^{125}$I TGFα bound (% Control) to:* Head and neck (HN5) | *Vulva (A431)* | *Breast (MDAMB468)* | *Bladder (EJ)* | *Ovarian (SKOV3)* |
|---|---|---|---|---|---|
| ICR60 | 41.8 | 31.6 | 19.9 | 14.0 | 34.9 |
| ICR61 | 31.3 | 25.8 | 12.9 | 10.4 | 6.9 |
| ICR62 | 24.5 | 24.1 | 10.3 | 7.2 | 6.4 |
| ICR63 | 38.3 | 24.1 | 12.5 | 4.6 | 20.3 |
| ICR64 | 15.4 | 18.7 | 4.8 | 6.4 | 7.4 |
| ICR65 | 33.4 | 25.8 | 9.4 | 5.6 | 4.3 |

**Figure 2** Competitive binding assays show that the rate antibodies recognise two distinct epitopes on the external domain of the EGFR. MAbs ICR60 (●), ICR61 (■), ICR62 (▲), ICR63 (▼), ICR64 (◆) and ICR65 (*) were competed with $^{125}$I-ICR63 or $^{125}$I-ICR64 for binding to monolayers of EJ cells.

**Figure 3** Autoradiographs of immunoprecipitates prepared from $^{35}$S-methionine-labelled cells, HN5 (top) or SKOV3 (bottom), and run on 7.5% reducing gels. Immunoprecipitates were prepared with mAbs to the EGF receptor (ICR10, ICR60–64), the product of the c-erbB-2 p185 (ICR12) or control antibody (ALN/11/53).

affinity and two of the Scatchard plots (ICR61, ICR64) showed two affinities for binding (Table I).

All six antibodies inhibited the binding of EGF and TGFα to the receptor present on five human tumour cell lines of different origin (Table II) and as expected maximal inhibition was observed with the cell lines (e.g. EJ and SKOV3) that expressed lower levels of the EGFR. This result suggests that most of the antibodies bind at or near to the ligand binding site on the receptor. However, the antibodies did not bind to the mouse EGFR since none of them inhibited the binding of $^{125}$I-EGF to the mouse cell line Ca6 (data not shown).

### *The antibodies do not crossreact with the product of the c-erbB-2 proto-oncogene*

MAbs ICR60–ICR64 and ICR10 (positive control antibody raised against EGFR on HN5 cells) all specifically immunoprecipitated the 170 kDa EGFR from detergent extracts of $^{35}$S-methionine-labelled HN5 cells whereas a control antibody (ALN/11/53) did not (Figure 3). The mAbs also immunoprecipitated the EGFR from detergent extracts of $^{35}$S-methionine-labelled SKOV3 cells but not the 185 kDa overexpressed product of the c-erbB-2 gene. The latter was, however, precipitated from these extracts (Figure 3) by antibody ICR12 (Styles *et al.*, 1990). We conclude that none of the antibodies tested (ICR60–64) crossreacted with the c-erbB-2 gene product.

### *Effect of antibody and or TGFα on proliferation of human fibroblasts*

Both EGF and TGFα stimulate DNA synthesis in quiescent human fibroblasts. We have investigated the effect of our antibodies used alone or in combination with growth factors on DE532 cells. The results, presented in Table III, show that none of the antibodies alone could stimulate DNA synthesis in these cells. All of the antibodies blocked TGFα stimulated DNA synthesis; completely in the case of ICR61–ICR64, but antibody ICR60 was less effectives. We conclude that all the

**Table III** Effect of anti-EGFR monoclonal antibodies, TGFα or both on DNA synthesis in quiescent human foreskin fibroblasts (DE532)

| | *$^{3}$H-Thymidine incorporation (CPM)* | | | | | | |
|---|---|---|---|---|---|---|---|
| *TGFα (ng ml$^{-1}$)* | *ICR60 25 μg ml$^{-1}$* | *ICR61 25 μg ml$^{-1}$* | *ICR62 25 μg ml$^{-1}$* | *ICR63 25 μg ml$^{-1}$* | *ICR64 25 μg ml$^{-1}$* | *ALN11/53 25 μg ml$^{-1}$* | *NO Antibody* |
| 0 | 240 | 201 | 247 | 250 | 199 | 223 | 316 |
| 5 | 770 | 189 | 190 | 308 | 221 | 1556 | 1755 |

antibodies acted as antagonists of TGFα. Similar results were obtained when EGF was used instead of TGFα (data not shown).

*The mAbs inhibit the growth of tumour cells that overexpress the EGFR*

The growth of HN5 and HN6 cells was inhibited completely when cultures were incubated with the antibodies to the EGFR at a concentration of 25 μg $ml^{-1}$ or greater (Table IV). When tested at lower concentrations, some of the mAbs were found to be more effective than others (Figure 4) and the order of effectiveness for inhibition of growth of HN5 cells was ICR64 (IgG1) > ICR62 (IgG2b) > ICR63 (IgG2a) > ICR60 (IgA) ≥ ICR61 (IgG2b). Antibodies ICR60–64 were also tested for their effect on the growth of a number of other tumour cell lines including those (A431, HN15, MDA-MB 468) with high levels of expression of the EGFR, or those (EJ, SKOV3, SKBR3) with lower levels of EGFR expression ($< 1 \times 10^5$ receptors/cell) or cells that did not express detectable levels of the EGFR (MDA-MB 435). The results of this comparative study using media containing 2% FCS are presented in Table IV. They show that growth of the cell lines with high level expression of the EGFR was inhibited by all of the antibodies tested. At a concentration of 156 nM, however, the antibodies were without effect on the growth of SKBR3, SKOV3, EJ or MDA-MB 435 cells.

To investigate if endogenous growth factors present in FCS affected the activity of the anti-EGFR antibodies, experiments were repeated using HN5 cells incubated in media containing 5% or 10% FCS. Only a small shift in baseline (10%) was observed with these antibodies (cf ICR64, Figure 5) even at the highest concentration of FCS used and we conclude that at the concentration present in FCS such growth factors had little effect on the growth inhibitory activity of the mAbs.

**Table IV** Effect of mAbs to the EGF receptor on the growth of tumour cells in medium containing 2% foetal calf serum

| mAbs (156nM) | Growth (% medium control) of: HN15 | HN5 | HN6 | A431 | MDA-MB468 |
|---|---|---|---|---|---|
| ICR60 | 39.6 | 2.7 | ND | ND | ND |
| ICR61 | 32.4 | −1.0 | −6.7 | 66.1 | 40.1 |
| ICR62 | 18.3 | 1.1 | −7.1 | 65.3 | 32.8 |
| ICR63 | −0.1 | −1.4 | 3.4 | 71.8 | 67.0 |
| ICR64 | 22.8 | 0.1 | 0.0 | ND | 39.7 |

No effect on SKBR3, SKOV3 and EJ cells.
ND = Not determined

**Figure 4** Influence of antibodies to the human EGFR on the growth of HN5 cells in DMEM containing 2% FCS. (▲), ICR60; (▼), ICR61; (◆), ICR62; (*), ICR63; (●), ICR64; (■), control ALN/11/53.

**Figure 5** Influence of the concentration of foetal calf serum on the inhibition of growth of HN5 cells by antibody ICR64. FCS concentration: (■), 2% (▲), 5%; (▼), 10%).

While the head and neck carcinomas HN5, HN6 and HN15 were the most sensitive to antibody induced inhibition of growth, A431 and MDA-MB 468 were less susceptible. A431 cells have been reported to secrete large amounts of a truncated form of the receptor (Weber *et al.*, 1984) and this may have influenced the interaction of the rat antibodies with the transmembrane receptor.

*The mAbs bind to the truncated EGFR secreted by A431 cells*

When $^{125}$I-labelled mAbs ($2.2–2.5 \times 10^4$) were mixed with culture supernatant obtained from A431 cells the binding of antibodies ICR60–64 to EJ cells was abolished (Table V). Also, the 100 kD truncated receptor was precipitated when cell free supernatants from $^{35}$S-methionine labelled A431 cultures were immunoprecipitated with mAbs ICR16 and ICR64 (Figure 6) but not when the control antibodies (ALN/11/53, ICR12, ICR55) were used. Clearly, with tumours like A431, the presence of large amounts of truncated receptor may decrease the effective dose of antibody reaching the tumour cells.

### Discussion

In this paper we describe the production and some of the properties of six new rat monoclonal antibodies directed against the human EGF receptor. Our results show that these antibodies have the following properties, they: (a) bind with high affinity to two distinct epitopes on the extracellular domain of the human EGFR, (b) do not cross-react with the related product of the c-erbB-2 proto-oncogene, (c) prevent the binding of exogenous growth factors to the receptor, (d) inhibit the growth stimulatory effects of EGF and TGFα on human fibroblasts and (e) inhibit the growth of human tumour cell lines overexpressing this receptor. Three of these antibodies are of the IgG2b isotype (ICR62, epitope C; ICR61, epitope D and ICR65, epitopes C and D). While all of the antibodies stained frozen sections of cells overexpressing the EGFR, none was found to bind the fully reduced

**Table V** Effect of supernatant from human epidermoid carcinoma cell line (A431) on the binding of $^{125}$I anti-EGF receptor mAb to the EGF receptor on EJ cells. Each value is the mean of triplicate samples

| Treatment | $^{125}$I Antibody Bound (cpm). ICR61 | ICR62 | ICR63 | ICR64 |
|---|---|---|---|---|
| A431 supernatant | 88 | 92 | 165 | 139 |
| Control medium | 1200 | 700 | 1289 | 2567 |

**Figure 6** Autoradiographs of immunoprecipitates of $^{35}S$-methionine labelled EGFR and truncated EGFR secreted by A431 cells run on 7.5% reducing gels. Track A: ICR16; track B ICR64; track C ICR12; track D ICR55; track E ALN/11/53; track F medium control.

receptor in Western blots nor did they bind to formol-saline fixed paraffin embedded tissues (data not shown). We conclude that mAbs ICR60–64 all recognise conformational determinants on the receptor.

During the last decade a number of laboratories have generated antibodies against the human EGFR with a view to their use in structural studies on the receptor or for clinical applications in oncology. In most cases the antibodies were raised in mice against the EGFR on the vulval carcinoma cell line A431. Because the A431 receptor also contains the blood group A antigen, many of the antibodies produced crossreact with this normal antigen limiting their use for diagnostic or therapeutic purposes (see review by Sato *et al.*, 1987).

As in A431 cells, the receptor for the EGFR is overexpressed on the surface of MDA-MB 468 cells but, unlike A431 cells, the gene for the EGFR is not rearranged (Filmus *et al.*, 1987). Our aim has been to produce rat monoclonal antibodies that bind to different epitopes on the external domain of the human receptor for EGF so that we can identify the best epitope to target for growth inhibition as well as the best antibody for harnessing host immune effector functions. For this reason we have used rats as our source of antibodies rather than mice because some rat antibodies of the IgG2b isotype interact very efficiently with the effector arm of the human immune system (Dyer *et al.*, 1989).

We have reported (Modjtahedi *et al.*, 1992) that rat mAbs raised against the head and neck carcinoma cell line LICR-LON-HN5 bound with high affinity to one of three distinct epitopes (A,B,C) on the EGFR but none were of the IgG2b isotype. Outstanding, in terms of inhibition of growth of HN5 cells, were antibodies ICR11 and ICR16 both of which were directed against epitope C. Here we report that two out of a further six mAbs raised against the EGFR on MDA-MB 468 cells also bound to epitope C and one of these was an IgG2b (ICR62) with good growth inhibitory properties *in vitro*. Interestingly, three of the four other mAbs recognised a new epitope (D) on the EGFR and one of these mAbs (ICR61) was an IgG2b. Lastly, antibody ICR65, which is of recent production, is also an IgG2b and this mAb cross-reacted with both epitopes C and D. The other properties of this antibody are currently being determined.

The isolation of three antibodies against a novel epitope on the EGFR was surprising since no antibodies of this type were obtained in nine separate fusions when HN5 cells had been used a immunogen. Clearly, this resulted from the use of a different source of EGFR as immunogen (MDA-MB468) which in turn led also to the isolation of three mAbs of the IgG2b isotype. These findings reinforce our view that it is essential to use different source of immunogen to obtain as diverse a population of antibodies (epitope/isotype) from which the best antibody for clinical use can be selected.

In terms of biological activity, antibody ICR61 was the least effective of the new antibodies and complete inhibition of growth of HN5 cells was only obtained at a concentration of 20 nM which was an order of magnitude higher than that required with ICR62 or ICR64, which inhibited completely the growth of these cells at a concentration of 2.4 nM. Indeed ICR64 was one of the best of all the rat mAbs tested and it equalled ICR16 (Modjtahedi *et al.*, 1992), in terms of its capacity to block the binding of EGF to its receptor and to inhibit the growth of HN5 cells *in vitro*. We conclude that both epitopes C and D constitute suitable targets for antibody directed therapy.

The antibodies very effectively inhibited the growth *in vitro* of three different head and neck carcinoma cell lines. They were less effective, however, when tested on A431 or MDA-MB 468 cells. The A431 cell line is aberrant in that it secretes large amounts of a truncated form of the EGFR (Weber *et al.*, 1984) to which all of the rat antibodies bind. This soluble EGFR may therefore reduce the effectiveness of the mAbs at inhibiting growth of this cell line. Furthermore, it has been shown that, in addition to overexpression of the EGFR, both A431 and MDA-MB 468 cells produce TGFα constitutively (Derynck *et al.*, 1987; Ennis *et al.*, 1989). High level secretion of this ligand by these cells may also reduce the effectiveness of the antibodies *in vitro* by competing with them for binding to the receptors and this aspect is under investigation.

Currently, we are testing some of the antibodies for their activity *in vivo* against xenografted human tumours growing in athymic mice and the results obtained are very encouraging. These experiments are described in the accompanying paper and show that ICR62 and ICR64 were effective at eliminating the tumours when given at the time of tumour cell inoculation. ICR62 was also effective in inducing regression of established tumours.

We believe that this new generation of rat mAbs which bind with high affinity to the EGFR, block ligand-receptor interaction and inhibit the growth of squamous cell carcinomas overexpressing the receptor have potential for clinical application.

This work was supported by a Programme Grant from the Cancer Research Campaign, London.

### References

BAZIN, H., XHURDEBISE, L.M., BURTONBOY, G., LEBACQ, A.M. DE CLERQ, L & CORMONT, F. (1984). Rat monoclonal antibodies. I. Rapid purification from *in vitro* culture supernatants. *J. Immunol. Methods*, **66**, 261–269.

COWLEY, G., SMITH, J.A., GUSTERSON, B., HENDLER, F. & OZANNE, B. (1984). The amount of EGF receptor is elevated on squamous cell carcinomas. *Cancer Cells*, **1**, 5–10.

DEAN, C.J., STYLES, J.M., GYURE, L.A., PEPPARD, J., HOBBS, S.M., JACKSON, E. & HALL, J.G. (1984). The production of hybridomas from the gut associated lymphoid tissue of tumour bearing rats. I. Mesenteric nodes as a source of IgG producing cells. Clin. Exp. *Immunol.*, **57**, 358–364.

DERYNCK, K., GOEDDEL, D.V., ULLRICH, A., GUTTERMAN, J.U., WILLIAM, R.D., BRINGMAN, T.S. & BERGER, W.H. (1987). Synthesis of messenger RNAs for transforming growth factor α and β and the epidermal growth factor receptor by human tumours. *Cancer Res.*, **47**, 707–712.

DERYNCK, R. (1990). Transforming Growth Factor-α. *Mol. Reprod. Develop.*, **27**, 3–9.

DIFIORE, P.P., PIERCE, J.H., HAZAN, R., ULLRICH, A., KING, C.R., SCHLESSINGER, J. & AARONSON, S.A. (1987). Overexpression of the human epidermal growth factor receptor confers a EGF-dependent transformed phase-type to NIH3T3 cells. *Cell*, **51**, 1063–1070.

DYER, M.J.S., HALE, G., HAYHOE, F.G.J. & WALDMAN, H. (1989). Effect of campath-I antibodies *in vivo* in patients with lymphoid malignancies: Influence of antibody isotype. *Blood*, **73**, 1431-1439.

ENNIS, B.W., VALVERIUS, E.M., BATES, S.E., LIPPMAN, M.E., BELLOT, F., KRIS, R., SCHLESSINGER, J., MASUI, H., GOLDENBERG, A., MENDELSOHN, J. & DICKINSON, R.B. (1989). Anti-epidermal growth factor receptor antibodies inhibit the autocrine-stimulated growth of MDA-468 human breast cancer cells. *Mol. Endocrinol.*, **3**, 1830-1838.

FENDLY, B.M., WINGET, M., HUDZIAK, R.M., LIPARI, M.T., NAPIER, M.A. & ULLRICH, A. (1990). Characterization of murine monoclonal antibodies reactive to either the human epidermal growth factor receptor or HER2/neu gene product. *Cancer Res.*, **50**, 1550-1558.

FILMUS, J., POLLAK, M.N., CAILLEAU, R. & BUICK, R.N. (1985). MDA-468, a human breast cancer cell line with a high number of epidermal growth factor (EGF) receptors, has an amplified EGF receptor gene and is growth inhibited by EGF. *Biochem. Biophys. Res. Comm.*, **128**, 898-905.

FILMUS, J., TRENT, J.M., POLLAK, M.N. & BUICK, R.N. (1987). Epidermal growth factor receptor gene-amplified MDA-468 breast cancer cell line and its nonamplified variants. *Mol. Cell. Biol.*, **7**, 251-257.

FRAKER, P.J. & SPECK, J.C. (1978). Protein and cell membrane iodination with sparingly soluble chloramide 1, 3, 4, 4-tetrachloro-3-6-diphenylglycouril. *Biochem. Biophys. Res. Comm.*, **80**, 849.

GULLICK, W.J., MARSDEN, J.J., WHITTLE, N., WARD, B., BOBROW, L. & WATERFIELD, M.D. (1986). Expression of epidermal growth factor receptors on human cervical, ovarian and vulval carcinomas. *Cancer Res.*, **46**, 285-292.

GULLICK, W.J. (1991). Prevalence of aberrant expression of the epidermal growth factor receptor in human cancers. *Br. Med. Bull.*, **47**, 87-98.

HALE, G., CLARK, M. & WALDMANN, H. (1985). Therapeutic potential of rat monoclonal antibodies: isotype specificity of antibody dependent cell mediated cytotoxicity with human lymphocytes. *J. Immunol.*, **134**, 3056-3061.

HARRIS, A.L. (1990). Epidermal growth factor receptor. (EGFr). Expression in human primary cancers. *Pro. Amer. Associ. Cancer Res.*, **31**, 458-460.

HENDLER, F.J. & OZANNE, B.W. (1984). Human squamous cell lung cancers express increased epidermal growth factor receptors. *J. clin. Invest.*, **74**, 647-651.

IMANISHI, K., YAMAGUCHI, K., HONDA, S. & ABE, K. (1988). TGFα alpha a possible autocrine growth factor for human adenocarcinoma of the lung. In Imuna H, Shizume K, Yoshida S (eds): *Progress in endocrinology*, Vol. 2 Amsterdam: Elsevier, pp. 1363-1368.

LINDMO, T., BOVEN, E., CUTTITTA, F., FEDORKO, J. & BUNN, J.P.A. (1984). Determination of the immunoreactive fraction of radiolabeled monoclonal antibodies by linear extrapolation to binding at infinite antigen excess. *J. Immunol. Methods*, **72**, 77-89.

LIVNEH, E., PRYWES, R., KASHLES, O., REISS, I.V., SASSON, Y.I., MORY, Y. & ULLRICH, A. (1966). Reconstitution of epidermal growth factor receptors in cultured hamster cells. *J. Biol. Chem.*, **261**, 12490-12497.

MENDELSOHN, J. (1989). Anti-EGF receptor monoclonal antibodies: Biological studies and potential clinical applications. *Trans. Amer. Clin. Climatol. Assoc.*, **100**, 31-38.

MODJTAHEDI, H., STYLES, J., BOX, G., ECCLES, S., GUSTERSON, B. & DEAN, C. (1982). Antitumour activity of rat Mabs to the human receptor for EGF. In *Mutant Oncogenes: Targets for Therapy*? Epenetos, A.A. & Lemoine, N.R. (eds). Chapman Hall (in press).

MORISHIGIE, K.I., KURACHI, H., AMEMIYA, K., FUJITA, Y., YAMAMOTO, T., MIYAKE, A. & TANIZAWA, O. (1991). Evidence for the involvement of TGFα and EGFR autocrine mechanism in primary human ovarian cancers *in vitro*. *Cancer Res.*, **51**, 5322-5328.

MURTHY, U., BASU, A., RODECK, U., HERLYN, M., ROSS, A.H. & DAS, M. (1987). Binding of an antagonistic monoclonal antibody to an intact and fragmented EGF-receptor polypeptide. *Arch. Biochim. Biophy.*, **225**, 549-560.

NISTAR, M., LIBERMANN, T.A., BETZHOLTZ, C., PETTERSSON, M., CLAESSON-WELSH, L.H., SCHLESSINGER, J. & WESTERMARK, B. (1988). Expression of messenger RNA's for platelete-derived growth factor and TGFα and their receptors in human malignant cell lines. *Cancer Res.*, **48**, 3910-3918.

OZANNE, B., RICHARDS, C.S., HENDLER, F., BURNS, D. & GUSTERSON, B. (1986). Overexpression of the EGF receptor is a hallmark of squamous cell carcinomas. *J. Pathol.*, **149**, 9-14.

PELLEGRINI, R., CENTIS, F., MARTIGNONE, MASTROIONNI, A., TAGLIBUE, E., TOSI, E., MENARD, S. & COLNAGHI, M.L. (1991). Characterization of a monoclonal antibody directed against the epidermal growth factor receptor binding site. *Cancer Immunol. Immunother.*, **34**, 37-42.

SAINSBURY, J.R.C., FARNDOWN, J.R., HARRIS, A.L. & SHERBERT, G.V. (1985) Epidermal growth factor receptors on human breast cancers. *Br. J. surg.*, **72**, 86.

SANTON, J.B., CRONIN, M.T., MACLEOD, C.L., MENDELSOHN, J., MASUI, H. & GILL, G.N. (1986). Effect of epidermal growth factor receptor concentration on tumorigenicity of A431 cells in nude mice. *Cancer Res.*, **46**, 4701-4705.

SATO, J.D., KAWAMOTO, T., LE, A.D., MENDELSOHN, J., POLIKOFF, J. & SATO, J.H. (1983). Biological effects *in vitro* of MAbs to human EGF receptors *Mol. Biol. Med.*, **1**, 511-514.

SATO, J.D., LE, A.D. & KAWAMOTO, T. (1987). Derivation and assay of biological effects of monoclonal antibodies to epidermal growth factor receptors. *Methods Enzymol.*, **146**, 63-81.

SCHREIBER, A.B., LASX, I., YARDEN, Y., ESHHAR, Z. & SCHLESSINGER, J. (1981). Monoclonal antibodies against receptor for epidermal growth factor induce early and delayed effects of epidermal growth factor. *Proc. Natl. Acad. Sci. USA*, **78**, 7535-7539.

SPORN, M.B. & ROBERTS, A.B. (1985). Autocrine growth factors and cancers. *Nature*, **313**, 745-747.

STYLES, J.M., HARRISON, S., GUSTERSON, B.A. & DEAN, C.J. (1990). Rat monoclonal antibodies to the external domain of the product of the CerbB-2 proto-oncogene. *Int. J. Cancer*, **45**, 320-324.

TATEISHI, M., ISHIDA, T., MITSUDOMI, T., KANEKO, S. & SUGIMECHI, K. (1990). Immunohistochemical evidence of autocrine growth factors in odenscarcinoma of the human lung. *Cancer Res.*, **50**, 7077-7080.

ULLRICH, A. & SCHLESSINGER, J. (1990). Signal transduction by receptors with tyrosine kinase activity. *Cell*, **61**, 203-212.

VELU, T.J., BEGUINOT, L., VASS, W.C., WILLINGHAM, M.C., MERLINO, G.T., PASTAN, I. & LAWY, D.R. (1987). Epidermal growth factor-dependent transformation by a human epidermal growth factor receptor proto-oncogene. *Science*, **238**, 1408-1410.

WATERFIELD, M.D., MAYES, E.L.V., STROOBANT, P., BENNET, P.L.P., YOUNG, S., GOODFELLOW, P.N., BANTING, G.S. & OZANNE, B.A. (1982). Monoclonal antibody to the human epidermal growth factor receptor. *J. Cell Biochem.*, **20**, 149-161.

WEBER, W., GILL, G.N. & SPIES, J. (1984). Production of an epidermal growth factor receptor-related protein. *Science*, **224**, 294-297.

YOSHIDA, K., KYO, E., TSUDA, T., TSUJINO, T., ITO, M. & TAHARA, E. (1990). EGF and TGFα, the ligand of hyperproduced EGFR in human esophageal carcinoma cells, act as a autocrine growth factor. *Int. J. Cancer*, **45**, 131-135.